# The Association between Daily Total Dietary Nutrient Intake and Recent Glycemic Control States of Non-Pregnant Adults 20+ Years Old from NHANES 1999–2018 (Except for 2003–2004)

**DOI:** 10.3390/nu13114168

**Published:** 2021-11-21

**Authors:** Yin Bai, Hao Zhang, Jie Yang, Lei Peng

**Affiliations:** 1 Department of Marketing, College of Management and Economics, Tianjin University, Tianjin 300072, China; baiyin@tju.edu.cn; 2 Department of Social Medicine and Health Education, School of Public Health, Peking University, Beijing 100000, China; 2011210118@stu.pku.edu.cn; 3 Public Health Clinical Center of Chengdu, Chengdu 610066, China; yangjieHJ@gmail.com; 4 Department of Epidemiology, School of Clinical Oncology, Peking University, Beijing 100143, China

**Keywords:** daily total intake of dietary nutrients, RGCS, HbA1c, NHANES, odds ratio

## Abstract

Background: Although daily total dietary nutrient intakes were potentially important factors in maintaining glycemic balance, their overall effect on glycemic control was still unclear among American adults. Objectives: We aimed to examine the association between daily total dietary nutrient intake and recent glycemic control status (RGCS). Methods: This cohort was composed of 41,302 individuals from the National Health and Nutrition Examination Survey (NHANES). The daily total intake of dietary nutrients and RGCS were independent and dependent variables, respectively. To evaluate their association, we carried out binary logistic regression, model fitting, linear discriminant analysis, and the receiver operator characteristic (ROC) analysis. Results: The result of robust check model showed that only the daily total dietary vitamin B6 intake (adjusted OR = 0.848; 95% CI: 0.738, 0.973; *p*-value = 0.019) was significantly negatively correlated with RGCS. When daily total dietary vitamin B6 intake and glycosylated hemoglobin (HbA1c) were used as independent variables and dependent variables, respectively, to fit the curves and lines, the established robust check model could distinguish American adults with different RGCS well. Moreover, the robust check model results of ROC analysis indicated that daily total dietary vitamin B6 intake might be a potential predictor for RGCS (AUC = 0.977; 95% CI: 0.974, 0.980; *p*-value < 0.001). Conclusions: This study showed that only daily total dietary vitamin B6 intake was a beneficial factor in RGCS, but it might need further multicenter or prospective studies to verify whether vitamin B6 had biological implications and public health meaning for glycemic control among American adults (specifically referred to non-pregnant participants over 20 years old).

## 1. Introduction

Dietary nutrients play an important role in maintaining the balance of blood glucose as a necessary substance to regulate the normal physiological function of the body, being roughly divided into macronutrients [1,2,3], dietary fiber (as an independent factor that distinguishes carbohydrates, included in this study) [4,5,6], minerals [7,8,9], and vitamins [10,11,12,13]. Many studies have shown that there was a positive correlation between recent glycemic control status (RGCS) and serum chromium, zinc, and magnesium levels [7,14,15,16,17,18,19,20]. Glycosylated hemoglobin (HbA1c), an irreversible product of blood glucose and hemoglobin, could provide information for long-term glycemic control [21]. Moreover, after the relationship between RGCS and HbA1c concentration was widely confirmed, the serum index was applied to diabetes diagnosis and glycemic monitoring practice [21,22]. Therefore, it was appropriate to use HbA1c as a predictor for RGCS among American adults (specifically referred to non-pregnant participants over 20 years old).

At present, most of the research conclusions on the association between daily total nutrient intake and RGCS have been one-sided. They did not analyze the overall effect of various nutrients on RGCS but analyzed minerals, vitamins, and macronutrients separately, which was not complete and systematic, and might have even led to obtaining inconsistent conclusions [23,24]. Although findings on the association between daily total dietary nutrient intake and RGCS were inconsistent and not enough to prove the relationship, these results, to a certain extent, could supply research hypotheses for future large-scale prospective or multi-center verification. Therefore, if we further explored the association between the adults’ RGCS and daily total dietary nutrient intakes, it was necessary to construct a holistic and optimal model to combine the macronutrients, minerals, and vitamins of daily total nutrient intake, as well as demographic characteristics, in order to draw a reliable conclusion. 

In addition, most studies on dietary factors affecting glycemic control have been conducted on diabetes patients [5,11,25,26,27], and therefore these conclusions could not be suitable for American adults to control blood glucose. Moreover, it is worth noting that insufficient sample size might also lead to biased conclusions, for instance, in the study of Intra et al. [26], the sample size of cases group was very small (cases group, n = 84; controls group, n = 2745), and therefore the results of this study might have a larger systematic bias. Therefore, we conducted the follow-up sampling survey study to estimate the association between daily total nutrient intake and RGCS among non-pregnant adults 20+ years old using a large-scale database from National Health and Nutrition Examination Survey (NHANES 1999–2018, except for 2003–2004).

## 2. Methods

### 2.1. Database and Study Population

We used the NHANES database, a nationally representative survey database collected biennially by the National Center for Health Statistics (NCHS), and employed a complex, multistage, probabilistic sampling design [28]. The database was publicly available on the Internet and can be downloaded by researchers around the world. All details about the database could be efficiently acquired at http://www.cdc.gov/nchs/nhanes/ (accessed on 27 May 2021), including relevant information such as strict quality control measures for the questionnaire data undertaken by NHANES. The 24 h dietary recall data from non-pregnant adults 20+ years of age participating in NHANES 1999–2002 and 2005–2018 surveys were followed biennially for all analyses. The database for analysis consisted of five parts: demographics data, dietary data, examination data, laboratory data, and questionnaire data. 

During the 1999–2018 NHANES survey cohorts, 101,316 preliminary participants were included in the study. Individuals without physical examination data (*n* = 2096), under 20 years of age (*n* = 47,208), pregnant (*n* = 2527), without an unusual diet compared food consumed yesterday and without reliable data (*n* = 6431), and 2003–2004 survey cycle data with the missing outcome variable (*n* = 1752) were excluded. Those with complete or reliable 24 h recall data (only day 1 data used) as judged by the United States Department of Agriculture’s (USDA) Food Surveys Research Group staff were included in the analyses. In addition, of the 41302 participants, 58 of the dietary survey data contained some missing indices (such as the intake of dietary fiber and folic acid), and we used the median to fill them. Finally, 41,302 subjects (20,458 males and 20,844 females, 50.0 ± 17.9 years for males and 50.2 ± 17.8 years for females) were certainly included in this study (Figure S1). All serum samples were collected under fasting condition. HCHS obtained the written informed consent from all participants and the ethical review committee approved all NHANES protocols.

### 2.2. Variables

In this study, the independent variables were daily total dietary nutrient intakes, containing protein, carbohydrate, total fat, dietary fiber, vitamin B1, vitamin B2, vitamin B6, total folate, vitamin B12, vitamin E, calcium, magnesium, iron, zinc, and copper. It is worth mentioning that the dietary energy value different from the category of dietary nutrients was also included in the follow-up analysis as an independent important parameter. The dependent variable was RGCS (HbA1c < 6.5% represents good RGCS, and HbA1c ≥ 6.5% represents poor RGCS). All variables involved in this study were divided into continuous variables and categorical variables. Continuous variables included energy, protein, carbohydrate, dietary fiber, total fat, total folate, vitamin B1, vitamin B2, vitamin B6, vitamin B12, vitamin E, calcium, magnesium, iron, zinc, copper, poverty income ratio (PIR), insulin, glucose, and hemoglobin. Categorical variables included gender, age, race, education level, body mass index (BMI), moderate or severe physical activity, hypertension, the doctor informing them they had diabetes, having at least 12 cups of alcoholic drink per year, consuming over 100 cigarettes in their lifetime, and adult food security. Details of all variable acquisition procedures can be found at http://www.cdc.gov/nchs/nhanes/.

### 2.3. Statistical Analysis

The results of normality test showed that it could not be considered that all continuous variables obeyed normal distribution. Therefore, in the stages of statistical description and single variable analysis, all continuous variables and categorical variables were expressed as median (25% percentile–75% percentile) and percentage (proportion), respectively. We used a nonparametric test (Mann–Whitney *U* test) for all continuous variables that did not obey normal distribution, as well as Pearson’s chi-squared test for all categorical variables. Then, in the multivariate analysis stage, we controlled different confounders and established four binary logistic regression models with the RGCS as the dependent variable to adjust the potential bias. Eventually, we performed model fitting with the HbA1c index as the dependent variable, and receiver operator characteristic (ROC) analysis was performed to calculate the area under the curve (AUC). The result of the collinearity diagnosis showed that there was no collinearity (variance inflation factor, VIF < 10) among the independent variables studied. Statistical significance was considered when *p*-value was below 0.05 (two-tailed). Data processing, statistical analysis, and graphic drawing were carried out with Stata version 13.1, IBM SPSS version 26.0, GraphPad Prism version 7.00, R version 4.0.2 (http://www.R-project.org, The R Foundation), and EmpowerStats software version 2.1 (http://www.empowerstats.com, X&Y Solutions, Inc., Boston, MA, USA).

## 3. Result

### 3.1. Baseline Characteristics

**Figure 1:**
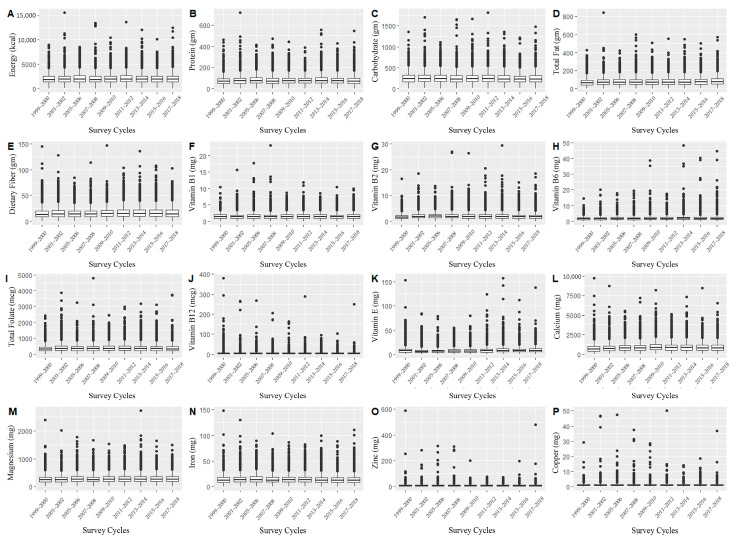
Distribution of daily total dietary energy and nutrient intakes in different investigation periods ((**A**) Energy (kcal); (**B**) Protein (gm); (**C**) Carbohydrate; (**D**) Total Fat (gm); (**E**) Dietary Fiber (gm); (**F**) Vitamin B1 (mg); (**G**) Vitamin B2 (mg); (**H**) Vitamin B6 (mg); (**I**) Total Folate (mg); (**J**) Vitamin B12 (mg); (**K**) Vitamin E (mg); (**L**) Calcium (mg); (**M**) Magnesium (mg); (**N**) Iron (mg); (**O**) Zinc (mg); (**P**) Copper (mg)).

The description of demographic and medical characteristics is shown in Table 1. Among the participants, 49.5% (*n* = 20,458) were male, 44.6% (*n* = 18,404) were non-Hispanic White, 20.5% (*n* = 8458) were non-Hispanic Black, and 17.3% (*n* = 7153) were Mexican American. In addition, the statistical description of daily dietary nutrient intakes in our study showed that their distribution fluctuated over time (Figure 1). Therefore, the time effect was often a potential confusion factor, which should be placed in subsequent analysis.

### 3.2. Binary Logistic Regression Analysis

#### 3.2.1. The Association between RGCS and Daily Total Dietary Energy, Macronutrients, Vitamins, and Minerals

**Figure 2:**
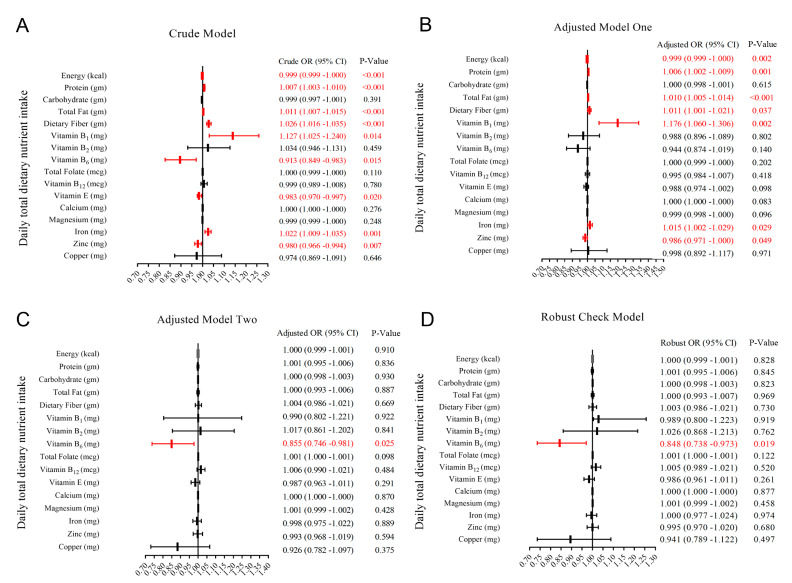
Forest plot for odds ratio (OR) and 95% confidence interval (CI) of daily total dietary nutrient and energy intake ((**A**) without covariates; (**B**) gender, age, and race were controlled; (**C**) all potential confounders in the study were controlled; (**D**) all potential confounders and the years fixed effect in the study were controlled).

For the crude model and adjusted model I, we found that energy, protein, total fat, dietary fiber, vitamin B1, vitamin B6, vitamin E, iron, and zinc intake were associated with RGCS, including good RGCS and poor RGCS (Figure 2A,B). However, after controlling of all covariates and time fixed effect (Figure 2C,D), opposite results were obtained from the former two models, for instance, three kinds of macronutrients, minerals, and dietary fiber actually had no statistical association with RGCS. Eventually, the statistical results in the robust check model (Figure 2D and Table 2), after controlling for the potential confounders and years fixed effect, suggested a significantly negative correlation between daily total dietary vitamin B6 intake and RGCS (adjusted OR = 0.848; 95% CI: 0.738, 0.973; *p*-value = 0.019). 

#### 3.2.2. The Association between Adjusted Covariates and RGCS

The statistical results of the robust check model, all covariates, and time fixed effect being adjusted demonstrated that age (taking “<40 years old” as a reference, OR_[40–59 years old]_ = 2.659, *p*-value < 0.001; OR_[≥60 years old]_ = 2.186, *p*-value < 0.001), gender (taking “female” as a reference, OR_[Male]_ = 1.365, *p*-value = 0.008), race (taking “other races” as a reference, OR_[Mexican American]_ = 0.796, *p*-value = 0.131; OR_[Non-Hispanic Black]_ = 1.486, *p*-value = 0.016; OR_[Non-Hispanic White]_ = 1.044, *p*-value = 0.804), education level (taking “>high school” as a reference, OR_[≤High School]_ = 1.205, *p*-value = 0.083), BMI (taking “<25.0” as a reference, OR_[25.0–29.9]_ = 2.042, *p*-value < 0.001; OR_[≥30.0]_ = 1.079, *p*-value = 0.626), hypertension (taking “no” as a reference, OR_[Yes]_ = 1.381, *p*-value = 0.001), the doctor informing them that they had diabetes (taking “no” as a reference, OR_[Borderline]_ = 12.072, *p*-value < 0.001; OR_[Yes]_ = 3.130, *p*-value < 0.001), insulin (OR = 1.003, *p*-value = 0.132), glucose (OR = 1.063, *p*-value < 0.001), and hemoglobin (OR = 0.918, *p*-value = 0.017) were significantly associated with RGCS in Table 2.

### 3.3. Model Fitting, Linear Discriminant Analysis, and ROC Analysis

#### 3.3.1. Model Fitting and Linear Discriminant Analysis of Daily Total Dietary Vitamin B6 Intake, Glycohemoglobin, and RGCS

**Figure 3:**
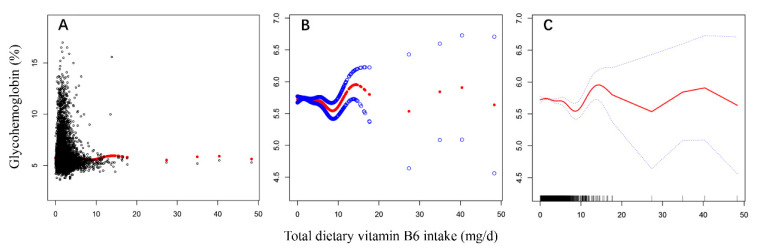
The model fitting processes of non-linear regression curve ((**A**,**B**), scatter plots; (**C**), the optimal smooth curve).

**Figure 4:**
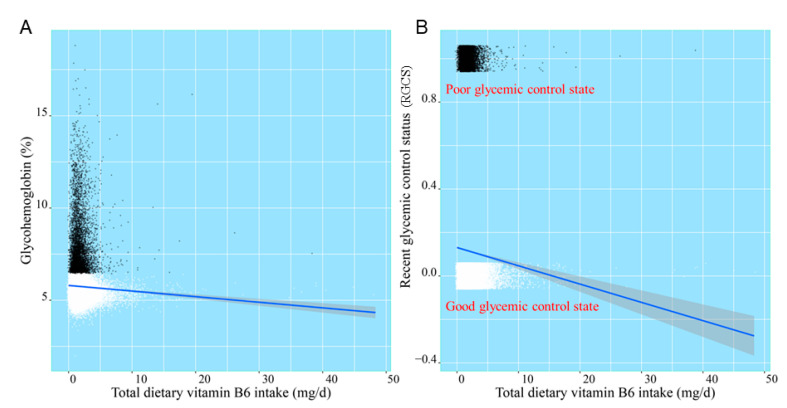
The linear discriminant analysis of daily total dietary vitamin B6 intake, glycohemoglobin (**A**), and RGCS (**B**).

After smooth curve fitting of daily total dietary vitamin B6 intake and glycohemoglobin being conducted in Figure 3, the robust check model, a linear discriminant analysis of daily total dietary vitamin B6 intake and RGCS, was also fitted in Figure 4. The statistical analysis graphs showed that the established robust check model could not only distinguish American adults with different RGCS well, but pointed out that the negative correlation between daily total dietary vitamin B6 intake and RGCS did exist. It was indicated that daily total dietary vitamin B6 intake might have a potential predictive value for RGCS of American adults.

#### 3.3.2. ROC Analysis of Daily Total Dietary Vitamin B6 Intake

**Figure 5:**
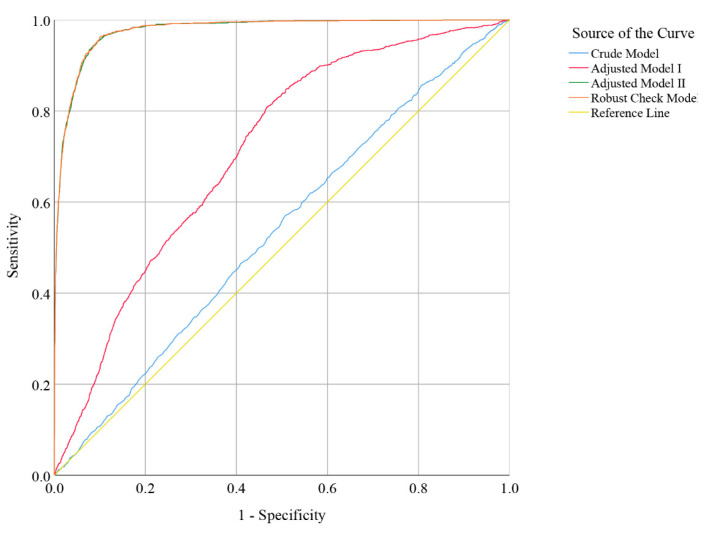
Receiver operator characteristic (ROC) curve indicator of poor RGCS (crude model: without covariates; adjusted model I: gender, age, and race were controlled; adjusted model II: all potential confounders in the study were controlled; robust check model: all potential confounders in the study were controlled, and the years fixed effect in the study was included).

ROC analysis of daily total dietary vitamin B6 intake was performed to calculate the area under the curve (AUC), which was used to evaluate the discrimination accuracy among people with good and poor RGCS. As shown in Figure 5, after controlling for all potential confounders and the years fixed effect, the predictive potential or accuracy of the multivariate logistic regression robust check model (AUC = 0.977; 95% CI: 0.974, 0.980; *p*-value < 0.001) was higher than those of the crude model (AUC = 0.535; 95% CI: 0.519, 0.550; *p*-value < 0.001), adjusted model I (AUC = 0.710; 95% CI: 0.697, 0.723; *p*-value < 0.001), and adjusted model II (AUC = 0.975; 95% CI: 0.974, 0.979; *p*-value < 0.001). The test results of DeLong between robust check model and crude model, and adjusted model I and adjusted model II showed that there were two statistically significant results (robust check model vs. crude model, *Z* = 53.54, *p*-value < 0.001; robust check model vs. adjusted model I, *Z* = 39.542, *p*-value < 0.001; robust check model vs. adjusted model II, *Z* = 0.751, *p*-value = 0.452).

## 4. Discussion

Although the reliable data of our study were from the national representative sample published by the Centers for Disease Control and Prevention of the United States, there were still some missing values in our collected data, which might have a subtle influence on our results of the statistical analysis. However, given the sufficient sample size of this study, the deviation caused by missing values could be reduced. Therefore, the reliability and authenticity of our findings were within acceptable limits. In addition, we did not ignore the interactions among nutrients when fitting the saturation model, but these interactions that might have biological significance (such as the interaction between vitamin B6 and vitamin B12) were not statistically significant when they were included in the robust check model.

The statistical model constructed in our research combined the specific macronutrients, minerals, vitamins, dietary fiber, and energy of the daily total diet, demographic, and medical indicators of American adults, because some statistical models mentioned in other studies might only be relevant for a small group of people with diabetes and not be suitable for American adults in terms of predicting their RGCS. Thus, our robust check model was closer to the real-world results than the models established by those research institutes [27,29]. 

Eventually, we found only daily total dietary vitamin B6 intake negatively correlated with RGCS, that is, the higher the daily total intake of dietary vitamin B6 was accompanied with better RGCS. Similarly, Mascolo et al. also concluded that the vitamin B6 level was significantly negatively associated with diabetes mellitus in diabetic people and suggested that vitamin B6 had a significantly protective effect on diabetic complications [30]. In addition, although covariates adjusted in our robust check model could not answer our research issues, they could still provide the theoretical foundation and scientific guidance for our health education related to glycemic control for American adults, which had a valuable public health significance.

The establishment of the robust check model of nutrients and RGCS was only the preliminary step of this study, which was mainly used to qualitatively find the associated factors affecting the RGCS. After screening the statistically significant daily total dietary vitamin B6 intake with this model, we further performed linear discriminant model and ROC analysis between RGCS and daily total dietary vitamin B6 intake as well as HbA1c, respectively (Figure 4 and Figure 5), which could provide a quantitative reference and prediction accuracy of daily total dietary vitamin B6 intake for American adults who need to control blood glucose.

Vitamin B6 was an intriguing molecule that was involved in a wide range of metabolic, physiological, and developmental processes. Its active form, 5’-pyridoxal phosphate (PLP), was a co-factor for approximately 150 metabolic responses to glucose, lipid, amino acids, DNA, and neurotransmitters [30,31,32,33,34]. These studies showed that vitamin B6 had a potential to regulate body metabolism (including blood glucose). Although the United States, South Korea, and Japan published the recommended total dietary intake of vitamin B6 (fluctuating around 1.1~1.6 mg/d) for specific populations [35,36,37], it might not be suitable for American adults who need to control blood glucose. Therefore, it was necessary for the relevant health management agencies in the United States to formulate the recommended value of the total daily dietary vitamin B6 intake of RGC for American adults, so as to provide an effective way for them to obtain better RGCS. However, the formulation of total daily dietary vitamin B6 recommended intake remains to be further explored.

## 5. Conclusions

In summary, our results indicated that only daily total dietary vitamin B6 intake was significant negatively associated with RGCS among all dietary nutrients we studied. Although this study provided a ROC prediction result of daily total dietary vitamin B6 intake for RGCS, we might require further validation of whether it would have a positive and effective preventive effect and biological implications on RGCS of American adults. 

## Figures and Tables

**Table 1:** Characteristics by RGCS of non-pregnant adults 20+ years old from NHANES 1999–2018 (except for 2003–2004).

Characteristics	Good RGCS (HbA1 < 6.5%) ^#^ *n* = 36,594	Poor RGCS (HbA1 ≥ 6.5%) ^#^ *n* = 4708	$\chi^{2}/Z\;\mathrm{Value}$	p-Value
Gender (%)			21.006	<0.001
Male	17,978 (87.9)	2480 (12.1)		
Female	18,616 (89.3)	2228 (10.7)		
Age (%)			2119.291	<0.001
≥60 years old	11,414 (80.1)	2843 (19.9)		
40–59 years old	12,125 (88.6)	1554 (11.4)		
<40 years old	13,055 (97.7)	311 (2.3)		
Race (%)			322.688	<0.001
Non-Hispanic White	16,857 (91.6)	1547 (8.4)		
Non-Hispanic Black	7201 (85.1)	1257 (14.9)		
Mexican American	6140 (85.8)	1013 (14.2)		
Other Races	6396 (87.8)	891 (12.2)		
Education level (%) ^†^			255.723	<0.001
≤High School	17,597 (86.1)	2845 (13.9)		
College or above	18,955 (91.1)	1855 (8.9)		
BMI (Kg/m^2^) *^†^			219.213	<0.001
≥30.0	102 (87.9)	14 (12.1)		
25.0–29.9	12,455 (90.5)	1304 (9.5)		
<25.0	11,298 (95.3)	557 (4.7)		
Moderate/severe physical activity (%) ^†^			162.861	<0.001
Yes	14,859 (91.1)	1457 (8.9)		
No	21,719 (87.0)	3249 (13.0)		
Hypertension (%) ^†^			537.915	<0.001
Yes	14,672 (84.7)	2648 (15.3)		
No	19,138 (92.2)	1611 (7.8)		
The doctor told you that you had diabetes (%)			18,424.978	<0.001
Yes	1711 (33.1)	3462 (66.9)		
Borderline	676 (76.2)	211 (23.8)		
No	34,207 (97.1)	1035 (2.9)		
Had at least 12 cups of alcoholic drink per year (%) ^‡†^			157.612	<0.001
Yes	25,714 (89.8)	2932 (10.2)		
No	8981 (85.2)	1559 (14.8)		
Consumed over 100 cigarettes in lifetime (%) ^†^			26.543	<0.001
Yes	16,589 (87.7)	2321 (12.3)		
No	19,979 (89.3)	2383 (10.7)		
Food security (%)			38.584	<0.001
Yes	26,893 (89.4)	3174 (10.6)		
No	5300 (86.7)	812 (13.3)		
PIR^ *†^	2.2 (1.2–4.2)	1.8 (1.0–3.3)	6.236	<0.001
Energy (kcal)	1948.0 (1441.1–2612.0)	1725.0 (1257.0–2329.0)	7.599	<0.001
Protein (gm)	72.3 (51.8–100.7)	69.0 (49.3–94.0)	3.809	<0.001
Carbohydrate (gm)	236.6 (170.6–319.7)	204.5 (147.4–278.1)	8.295	<0.001
Total fat (gm)	71.3 (47.6–102.3)	64.6 (42.7–96.2)	4.666	<0.001
Dietary fiber (gm)	14.3 (9.3–21.2)	14.1 (9.3–20.8)	1.140	0.148
Thiamin (Vitamin B1) (mg)	1.4 (1.0–2.0)	1.4 (0.9–1.9)	2.685	<0.001
Riboflavin (Vitamin B2) (mg)	1.8 (1.3–2.6)	1.7 (1.2–2.3)	4.151	<0.001
Vitamin B6 (mg)	1.7 (1.1–2.5)	1.6 (1.1–2.3)	4.416	<0.001
Total folate (mcg)	341.0 (230.0–496.0)	320.0 (215.0–459.0)	3.558	<0.001
Vitamin B12 (mcg)	3.7 (2.1–6.2)	3.4 (1.9–5.6)	3.452	<0.001
Vitamin E (mg)	6.5 (4.2–10.0)	6.1 (3.8–9.3)	3.294	<0.001
Calcium (mg)	779.0 (496.0–1151.0)	713.0 (468.0–1040.8)	4.185	<0.001
Magnesium (mg)	265.0 (190.0–363.0)	249.0 (180.0–336.0)	4.041	<0.001
Iron (mg)	12.8 (8.9–18.4)	12.3 (8.5–17.5)	2.466	<0.001
Zinc (mg)	9.7 (6.6–14.1)	8.9 (6.1–13.0)	3.824	<0.001
Copper (mg)	1.1 (0.8–1.5)	1.0 (0.7–1.4)	4.124	<0.001
Insulin (uU/mL) ^†^	9.5 (6.2–15.2)	14.7 (8.8–25.0)	10.705	<0.001
Glucose (mg/dL) ^†^	91.0 (85.0–99.0)	149.0 (118.0–149.0)	45.353	<0.001
Hemoglobin (g/dL) ^†^	14.2 (13.2–15.2)	13.9 (12.8–15.0)	5.840	<0.001

^#^ The statistical description of the two groups was expressed in the form of a number (%)/median (25% percentile–75% percentile). ^†^ There were missing values in the two groups. ^‡^ One cup of alcoholic drink is equivalent to 12 ounces of beer, 4 ounces of wine, or an ounce of liquor. * BMI, body mass index; PIR, poverty income ratio; RGCS, recent glycemic control states.

**Table 2:** Binary logistic regression analysis between RGCS and dietary nutrient and energy intake among non-pregnant adults 20+ years old from NHANES 1999–2018 (except for 2003–2004).

Variables	Crude Model ^a^	Model I ^b^	Model II ^c^	Robust Check Model ^d^
	*β* (*SE*)	*β* (*SE*)	*β* (*SE*)	*β* (*SE*)
Energy (kcal)	−0.001 (0.0002) ***	−0.001 (0.0002) **	−0.00003 (0.0003)	−0.00006 (0.0003)
Protein (gm)	0.007 (0.002) ***	0.006 (0.002) **	0.001 (0.003)	0.001 (0.003)
Carbohydrate (gm)	−0.001 (0.001)	−0.0005 (0.001)	0.0001 (0.001)	0.0003 (0.001)
Total fat (gm)	0.011 (0.002) ***	0.010 (0.002) ***	−0.0005 (0.003)	−0.0001 (0.003)
Dietary fiber (gm)	0.025 (0.005) ***	0.011 (0.005) *	0.004 (0.009)	0.003 (0.009)
Thiamin (Vitamin B1) (mg)	0.120 (0.049) *	0.162 (0.053) **	−0.011 (0.107)	−0.011 (0.108)
Riboflavin (Vitamin B2) (mg)	0.034 (0.045)	−0.012 (0.050)	0.017 (0.085)	0.026 (0.085)
Vitamin B6 (mg)	−0.091 (0.037) *	−0.058 (0.039)	−0.157 (0.070) *	−0.165 (0.070) *
Total folate (mcg)	−0.0004 (0.0002)	−0.0003 (0.0002)	0.001 (0.0004)	0.001 (0.0004)
Vitamin B12 (mcg)	−0.001 (0.005)	−0.005 (0.006)	0.006 (0.008)	0.005 (0.008)
Vitamin E (mg)	−0.017 (0.007) *	−0.012 (0.007)	−0.013 (0.013)	−0.014 (0.013)
Calcium (mg)	−0.00008 (0.00008)	0.0001 (0.00008)	0.00002 (0.0001)	−0.00002 (0.0001)
Magnesium (mg)	−0.001 (0.0005)	−0.001 (0.0005)	0.001 (0.001)	0.001 (0.001)
Iron (mg)	0.022 (0.006) **	0.015 (0.007) *	−0.002 (0.012)	0.0004 (0.012)
Zinc (mg)	−0.020 (0.007) **	−0.015 (0.007) *	−0.007 (0.013)	−0.005 (0.013)
Copper (mg)	−0.027 (0.058)	−0.002 (0.057)	−0.077 (0.086) **	−0.061 (0.090)
Age (<40 years old)	-	Reference	Reference	Reference
Age (40−59 years old)	-	2.211 (0.103) ***	0.985 (0.190) ***	0.978 (0.191) ***
Age (≥ 60 years old)	-	1.561 (0.104) ***	0.792 (0.189) ***	0.782 (0.189) ***
Gender (male)	-	0.230 (0.058) ***	0.322 (0.117) **	0.311 (0.118) **
Race (other races)	-	Reference	Reference	Reference
Race (Mexican American)	-	−0.551 (0.083) ***	−0.269 (0.150)	−0.229 (0.151)
Race (non-Hispanic Black)	-	0.298 (0.089) **	0.342 (0.163) *	0.396 (0.164) *
Race (non-Hispanic White)	-	0.209 (0.091) *	−0.024 (0.170)	0.043 (0.172)
Education level (≤ high school)	-	-	0.179 (0.107)	0.187 (0.108)
BMI ^†^ (<25.0)	-	-	Reference	Reference
BMI (25.0–29.9)	-	-	0.726 (0.146) ***	0.714 (0.146) ***
BMI (≥30.0)	-	-	0.081 (0.156)	0.076 (0.156)
Moderate/severe physical activity (no)	-	-	0.114 (0.103)	0.076 (0.104)
Hypertension (yes)	-	-	0.296 (0.100) **	0.323 (0.100)
The doctor told you that you had diabetes (no)	-	-	Reference	Reference
The doctor told you that you had diabetes (borderline)	-	-	2.501 (0.106) ***	2.491 (0.106) ***
The doctor told you that you had diabetes (yes)	-	-	1.167 (0.196) ***	1.141 (0.196) ***
Had at least 12 cups alcoholic drink per year (yes)	-	-	−0.057 (0.112)	−0.077 (0.113)
Consumed over 100 cigarettes in their lifetime (yes)	-	-	−0.085 (0.103)	−0.073 (0.104)
Food security (no)	-	-	0.076 (0.135)	0.047 (0.137)
PIR ^ †^	-	-	−0.049 (0.036)	−0.048 (0.036)
Insulin (uU/mL)	-	-	0.003 (0.002)	0.003 (0.002)
Glucose (mg/dL)	-	-	0.061 (0.002) ***	0.061 (0.002) ***
Hemoglobin (g/dL)	-	-	−0.095 (0.036) **	−0.086 (0.036) *
Years fixed effect	-	-	-	Included

^a^ A total of 15 dietary variables were entered in the crude model: protein, carbohydrate, total fat, dietary fiber, vitamin b1, vitamin b2, vitamin b6, total folate, vitamin b12, vitamin e, calcium, magnesium, iron, zinc, copper. ^b^ Three variables were adjusted in model I: gender, age, race. ^c^ A total of 17 variables were adjusted in model II: gender, age, race, education level, BMI, moderate or severe physical activity, hypertension, the doctor informing them that they had diabetes, having at least 12 cups of alcoholic drink per year, consuming over 100 cigarettes in their lifetime, food security, PIR, energy, insulin, glucose, hemoglobin. ^d^ Robust check model: Based on model II, years fixed effect was adjusted. ^*^ *p*-value < 0.05; ^**^ *p*-value < 0.01; ^***^ *p*-value < 0.001. ^†^ BMI, body mass index; PIR, poverty income ratio; RGCS, recent glycemic control states; SE, standard error.

## Data Availability

Data described in the manuscript, codebook, and analytic code will not be made available because the data used in this study were from the NHANES database, which is a free and open database for all researchers around the world.

## Supplementary Materials

The following are available online at https://www.mdpi.com/article/10.3390/nu13114168/s1, Figure S1: Participant flow chart.

## Abbreviations

AUC: area under the curve; BMI, body mass index; CI, confidence interval; HbA1c, glycosylated hemoglobin; NCHS, National Center for Health Statistics; NHANES, National Health and Nutrition Examination Survey; OR, odds ratio; PIR, poverty income ratio; RGCS, recent glycemic control status; ROC, receiver operator characteristic; SE, standard error; USDA, United States Department of Agriculture; VIF, variance inflation factor.
