# Supplementary figures and images for: The Association between Daily Total Dietary Nutrient Intake and Recent Glycemic Control States of Non-Pregnant Adults 20+ Years Old from NHANES 1999–2018 (Except for 2003–2004)
Source: Nutrients. 2021 Nov 21;13(11):4168. doi: 10.3390/nu13114168 (PMC8620762; doi:10.3390/nu13114168)

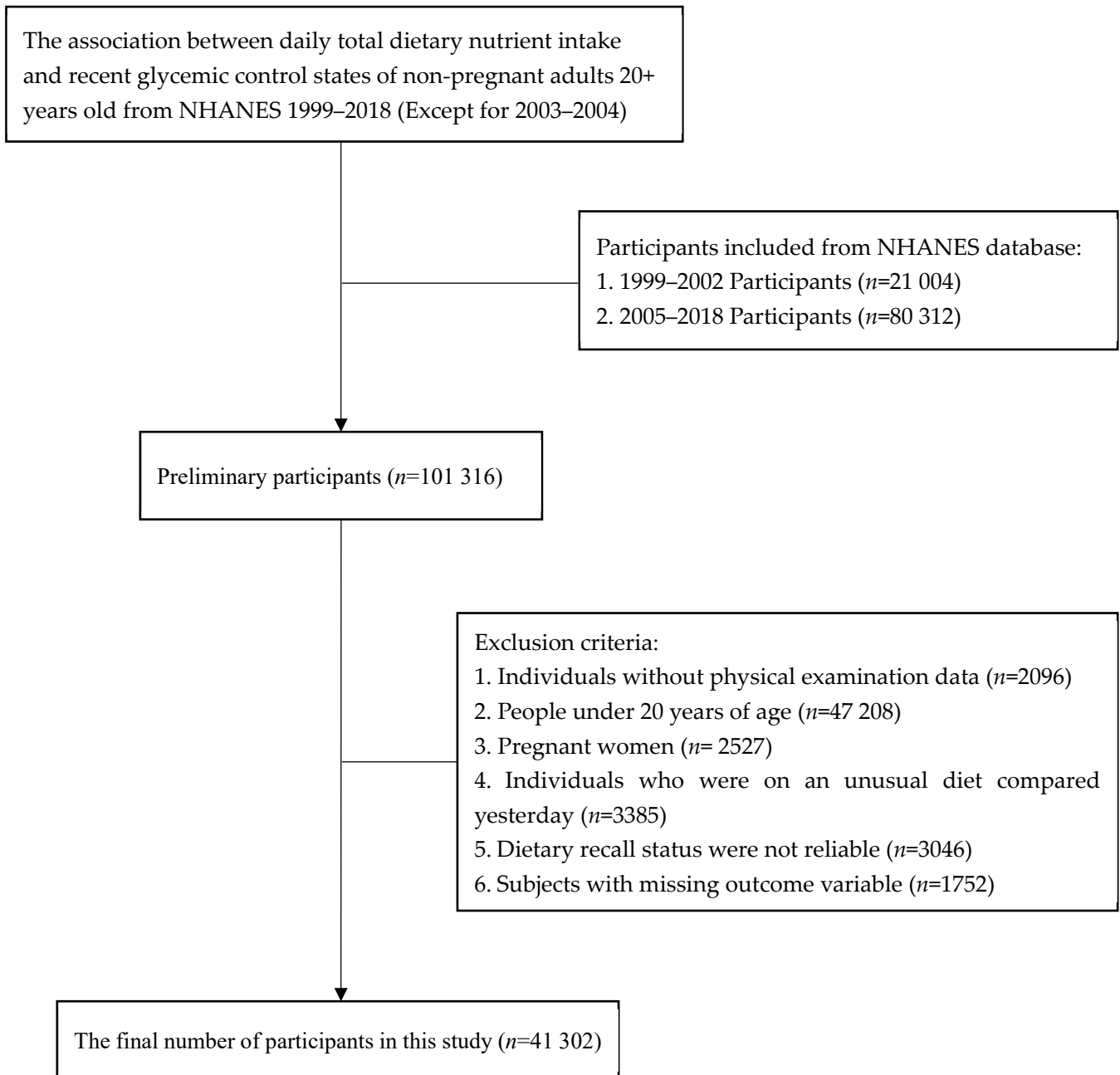

**Supplementary Figure S1.** Participant flow chart.

Supplement: Supplementary file 1 [file nutrients-13-04168-s001.zip › nutrients-1406925-supplementary.pdf]
